# Modified WHO Partograph in Labour Room: A Quality Improvement Initiative to Find Out Concerns, Challenges and Solutions

**DOI:** 10.7759/cureus.30851

**Published:** 2022-10-29

**Authors:** Ritu Singh, Mukta Agarwal, Sudwita Sinha, Hemali H Sinha, Monika Anant

**Affiliations:** 1 Obstetrics and Gynaecology, All India Institute of Medical Sciences, Patna, Patna, IND

**Keywords:** challenges, concerns, root cause analysis, partograph, quality improvement

## Abstract

Every day many women die in pregnancy and childbirth, most of which are preventable. Regular and timely labour monitoring by partograph is of utmost importance. The aim of this study was to increase partograph use by residents in the Department of Obstetrics and Gynecology in all eligible women from existing 25% to 90% over six months through a quality improvement (QI) process.

A team of six members including consultants, residents, and staff nurses did a root cause analysis through fishbone analysis to identify why the rate of use of partograph is only 25% of all cases. Many strategies were implemented through Plan-Do-Study-Act (PDSA) cycles for the cause identified. The interventions were allocation of triage area for timely identification of eligible women in the active phase of labour, training of residents, involving interns and nurses for use in shortage of staff, making departmental written policy, and assigning checking authority, to shift patients with attached partograph only; partograph has to be attached in the file right from the beginning when sisters make women admission file. These were done in five PDSA cycles and the outcome was measured by a control chart. The rate of partograph use increased from 25% to 92% over the study period of six months from September 2020 to February 2021. Regular audits were conducted to maintain the results. It can thus be concluded that partograph appears easy to implement and inexpensive, but its use still has enormous difficulties. But a QI approach can help in improving adherence to partograph use, by solving the root cause of the concern and challenges.

## Introduction

According to World Health Organization (WHO), in 2017, worldwide 295,000 women died in pregnancy and childbirth, approximately 810 women every day. Of these 94% occurred in low-middle-income countries, and the majority were preventable [1]. Women are picking healthcare facilities for delivery, as there is a global drive towards universal health coverage; but unless service quality is certain, health outcomes will not improve [2]. Monitoring of labour has been identified as a high priority for preventing maternal mortality by WHO [3].

For deciding on time, regular and timely labour monitoring is of utmost importance. WHO strongly recommends partograph use in the active management of labour [4]. But for record-keeping purposes, many healthcare workers complete partographs retrospectively [5]. Studies have mentioned how many people are using the partograph [6-8]. Some studies have found barriers to its use like lack of time [7], and shortage of staff [7,9,10]. However, only a few studies have been done on quality improvement for use of modified WHO partograph [11-13]. Thus, we have done a quality improvement initiative which is an initiative taken to improve the institution-specific processes [14]. This was done to find out concerning barriers, challenges in its use and solutions for regular and timely labour monitoring in the form of a partograph. The aim was to increase partograph use by residents in the Department of Obstetrics and Gynaecology in all eligible women from existing 25% to 90% over six months through a quality improvement (QI) process. The degree to which a change is implemented as intended (here 25% to 90%) is called fidelity. Fidelity is equivalent to treatment adherence in controlled clinical trials [15]. So, the more fidelity the better.

## Materials and methods

The quality improvement (QI) study was implemented to address the research question “Will the quality improvement process improve partograph use in all eligible women in the labour room of All India Institute of Medical Sciences (AIIMS) Patna from existing 25% to achieve 90% over six months duration?” after obtaining ethical approval from the Institutional Research Committee (Approval number- AIIMS/Pat/IRC/2020/513).

Study design

It was a quasi-experimental study. Pre-implementation and post-implementation mixed-methods study designs were used, in which observations are made before and after the implementation of an intervention. It is a mixed method because it combines elements of quantitative research and qualitative research. Fishbone analysis was done to find out the root cause of concerns and challenges. It was divided into four parts depending upon the procedure, people, policy, and place.

Hospital setting

AIIMS Patna is a prestigious institute in Eastern India. It is a tertiary care institute which provides essential and emergency obstetrics services [16,17]. There is one senior resident, three resident doctors in each 12-hour shift and three staff nurses on duty in each 8-hour shift, which makes a labour room team. The labour room team was using the modified WHO partographs [18] to monitor labour but had not received any formal training in QI methods.

Study participants

We have followed the following steps proposed by Etchells et al. [19] for sample size calculation in quality improvement initiatives:

(1) Define the eligible sample - We included all term singleton pregnancies in the active phase of labour.

(2) Establish exclusion criteria - Complicated pregnancies either obstetrics or medical were excluded from the study. Women who planned for elective caesarean section were also excluded.

(3) State the study period for each cycle/sample - 15 days to one month for each cycle in our study.

(4) Keep a reject log, and

(5) Ensure complete data collection.

We have aimed to enroll consecutive eligible patients. Random sampling is ideal but usually not practical.

Study procedure

**Figure 1:**
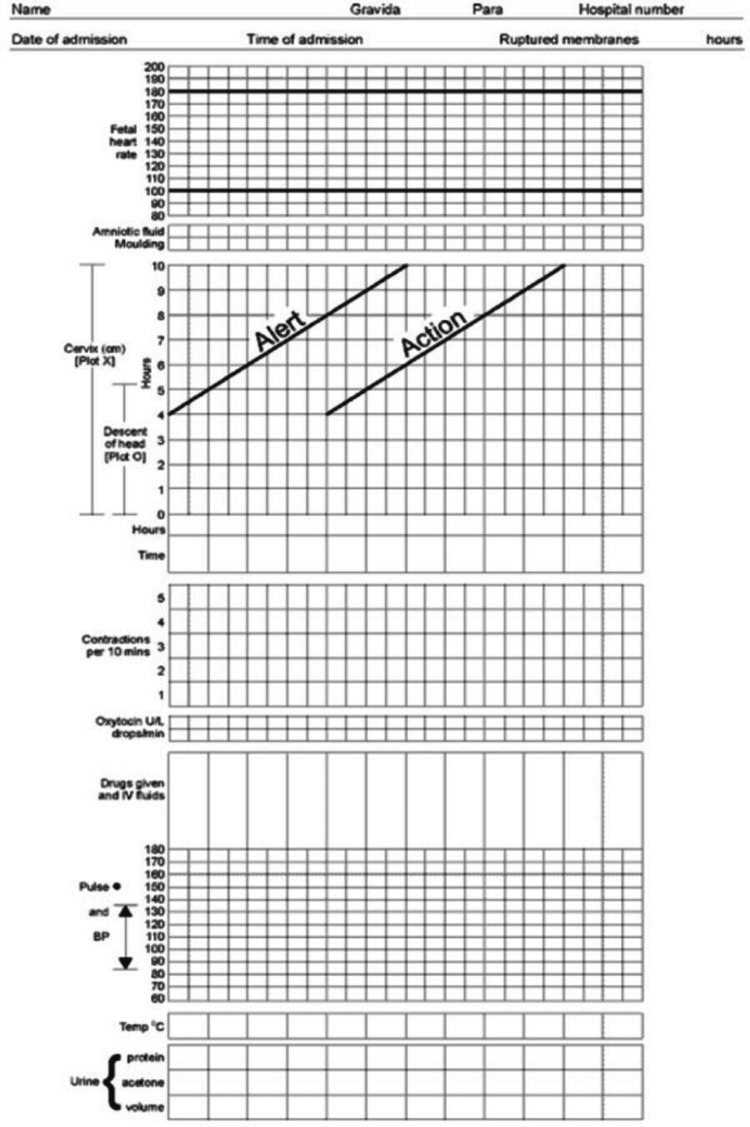
Modified WHO Partograph

The Labor Room Quality Improvement Initiative (LaQshya) programme [20] guidelines of intrapartum care by the Government of India were followed as it was a hospital policy. The study was carried out in the following steps:

1. A team of six healthcare providers was identified including consultants, resident doctors, and staff nurses (QI team). The team was decided on the basis of stakeholder mapping and analysis tools [21]. A stakeholder is anyone who has an interest in a project and can influence its success or failure [22]. We graphically used lines or arrows, made a stakeholder map with the quality problem remaining at the centre of the map and the different stakeholder groups organized around it. Then the analysis was done on which stakeholder to approach on the basis of the power versus interest grid [22]. These grids place stakeholders on a two-by-two matrix, where the y-axis is the stakeholder’s interest in the quality problem and the x-axis is the stakeholder’s organizational power or control over the system.

2. Baseline rates of partograph use in the labour room were measured over a week. Modified WHO partograph (Figure 1) is a labour monitoring tool proposed by the WHO [18] and also recommended by the WHO for use in active labour [4].

The modified WHO partograph is for plotting maternal and fetal conditions during the active stage of labour. It has three parts. The top part is for fetal conditions. Fetal heart rate is represented with dots and connected with dots. There is a place to fill the status of amniotic fluid and moulding. The middle part is for the progress of labour, and dilation of the cervix is plotted with respect to time. It has two diagonal lines, one alert and the other action. The bottom part is for maternal conditions like contraction, oxytocin, drugs, pulse, blood pressure, temperature, urine protein, urine volume, and urine acetone (Figure 1).

3. QI team met fortnightly with the labour room team to identify the root cause of poor compliance with partograph use.

4. Multiple Plan-Do-Study-Act (PDSA) cycles were conducted. Training of residents and interns on how to plot partograph was started in small group discussions in the labour room itself three days a week, so that every unit in the department gets its advantage.

5. Analysis of the data was done. Descriptive statistics were used to describe baseline variables. Control charts were used to display and interpret the serial measurement of indicators and to study the impact of changes.

Measuring parameter

The outcome measure was the percentage of eligible (women fulfilling inclusion criteria) women in whom the partograph was used. It was calculated by dividing the number of eligible women in active labour in whom the partograph was used (numerator) by the total number of eligible women in whom the partograph should have been used (denominator).

The percentage of partograph used = Number of partograph used multiplied by 100 / Total number of women in whom the partograph should have been used.

## Results

Identifying the problem (baseline assessment)

For a week, baseline data were collected. Every day a dedicated person assigned to work, examined case records of all women who had delivered a day before. The number of plotted partographs was noted in eligible women. The percentage was calculated by dividing the number of partograph used by the total number of women in whom the partograph should have been used and then multiplying it by 100. It came out that only 25% of women's partograph was plotted out of the total eligible women.

Identifying concerns and challenges

**Figure 2:**
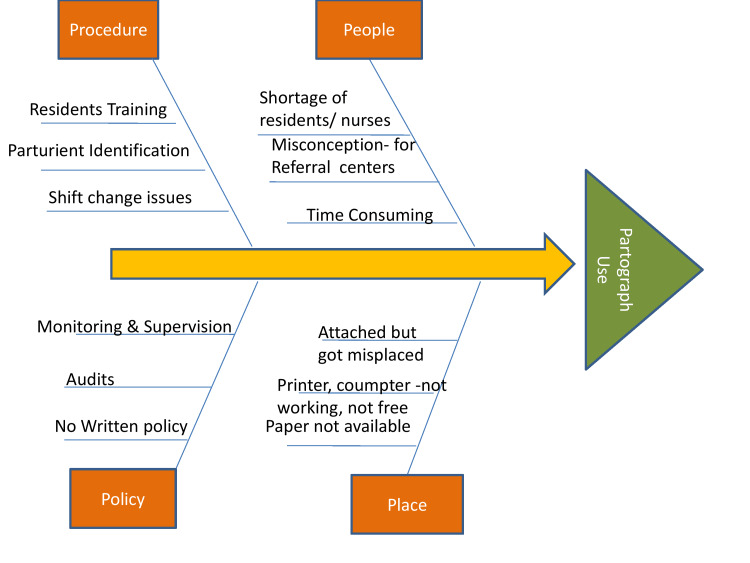
Various concerns and challenges identified by fishbone analysis

A session was conducted by the QI team. The various concerns and challenges identified by fishbone analysis are shown in Figure 2.

Strategy of action

After identifying different concerns and challenges with the help of fishbone analysis, the QI team planned PDSA cycles to execute various ideas.

PDSA Cycle I: Triage Room Allocation

When a woman came to the labour room after making admission through the outpatient department or directly on an emergency basis, it took approximately 1 hour for a senior resident to examine her, assign her stage of labour and plan for further management. The triage room was allotted just before the labour room where women were examined within 15 minutes after reporting to the labour room. Women in labour were kept in separate rooms so that their contraction and progress of labour could be plotted on time. In 15 days, partograph use had increased to 30%.

PDSA Cycle II: Training of Residents

**Figure 3:**
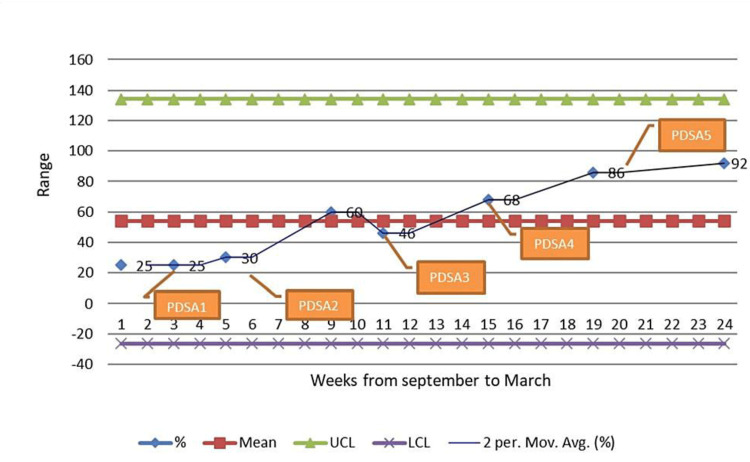
Control chart of percentage plotting (use) for partograph Mean = 54 UCL (Upper control limit) - three times of standard deviation away from mean = 134.3546 LCL (Lower control limit) - three times of standard deviation away from mean = -26.3546 PDSA - Plan-Do-Study-Act Cycle

The importance of partograph filling was emphasized. Residents' doubts were cleared, and misconception about the partograph that it is for the primary health care system for referring patients was cleared among residents. This cycle continued for one month which subsequently increased partograph use to 60% (Figure 3).

In the next 15 days, there was a fall in the use of partograph to 46% (Figure 3) because of an increase in the patient load as the festival season came and many private clinics were closed, and adding to it some of our trained labour room staff went on leave due to important festivals.

PDSA Cycle III: Involving Interns and Student Nurses, Handover Register

To overcome the overload of patients and shortage of staff, interns and student nurses were also trained for helping in partograph use. On further follow-up meetings, it was found that during shift change, the partograph was not plotted, so the handover register was maintained in the labour room in which patient details and partograph details were also noted. This increased partograph use to 68% (Figure 3).

PDSA Cycle IV: Written Policy and Assigning Checking Authority

The meeting was conducted with the head of the department and one departmental written policy was made for plotting of partograph. As in the labour room team, no one was taking responsibility for plotting partograph, senior resident in each shift was assigned as checking authority for partograph, and it was written clearly in the policy - without partographs patients cannot be shifted to another ward. Post-natal ward nurses had to take over the patients only if a partograph was present in the file. It led to a rise in partograph use to 86% (Figure 3).

PDSA Cycle V: Attaching Partograph in the File Right From the Beginning

Subsequently, it was found out that sometimes the printer was out of order, the partograph was not there, the labour room computer was busy, and the partograph was attached but it got misplaced. Our hospital has hospital information system software in which a computer is needed for raising investigation, for indenting drugs and raising dossier. So, it was decided by the QI team that a partograph had to be attached to the file right from the beginning when nurses make women's admission file. And also partograph had to be printed from the same printer from where other papers of admission files were being printed, labour room computer and printer were kept reserved for other works. At the end of the fifth PDSA cycle, the use rate increased from 86 to 92% with a 99.7% confidence interval of 108 (Figure 3).

Interns were assigned to make audits every 15 days and report back to the QI team. Every intern who came for posting in the labour room had to audit the partograph use. So, they were taking it seriously and learning beforehand how to fill the partograph. As the QI progressed, it was also added to the department policy that every six months, teaching and training will be conducted, when new residents will join the department.

Weekly plotting of the percentage of use of partograph on the control chart was done. The mean of the measured values was 54. The standard deviation (SD) came out to be 25.78486. Then upper control limit (UCL) was calculated by the formula UCL = Mean+(3xSD) which came 134.3546. The lower control limit (LCL) was calculated by the formula LCL = Mean-(3xSD) which came -26.3546. Then control chart was prepared using Microsoft Excel (Microsoft® Corp., Redmond, WA) (Figure 3).

## Discussion

The labor room of AIIMS, Patna saw a remarkable change in the use of partograph after the quality improvement study and the implementation of the change - from the initial 25% use of partograph to 92%. The concern and challenges behind its low usage according to different studies are availability or supply of partograph [7,9,10,23], time [7], shortage of staff [7,9,10], limited knowledge and understanding [7,9,10,24], lack of training [23] and confusion over roles and responsibilities [7,8]. These concerns and challenges were consistent with our study. When we go to a root level and do a PDSA cycle, we don’t need multiple studies to find barriers - one study is sufficient as ours. But how to find its solution is mentioned in limited studies [11-13], to the best of our literature review. According to Bedwell et al. [25] in a realistic review, ‘There is a limited description of implementation strategies in the reviewed literature’ but our study found the solution to these problems.

The solutions or the intervention we have done were allocations of a triage area for timely identification of eligible women in the active phase of labour, training of residents, involving interns and nurses for use in shortage of staff, making departmental written policy, and assigning checking authority, to shift patient with attached partograph only; partograph has to be attached in the file right from the beginning when sisters make women admission file. Partograph has to be printed from the same printer from where other papers of admission file are printed.

In the limited studies, a study done in Uganda [11] for improving partograph use in midwives has shown the solution for increased use of partograph is constant supervision, training, and making partograph available. The focal person was chosen to attach partograph to the admission file. These are consistent with our study but we have not chosen a focal person, instead, we have attached it to the file right from the beginning. Another study in 2020 [12] was conducted in a total of 141 facilities across 26 high-priority districts of India. They have also done interventions or found solutions by training healthcare workers; they have taken initiative so that prints of partograph are made available at all facilities. Measures were taken to have a perforated margin for the partograph so that it could be torn off easily during referral. Encouraging the practice of signing off, and handing over the partograph during shift change and monthly audits helped in coordination between doctors and nurses. These are consistent with our study. We have not used perforated margins as we don’t have to refer patients. Another quality improvement study was done by Jain et al. in 2022 [13] in a tertiary care centre for using e-partograph. They have also focused on the training, allocation of triage, and assigning responsibility to sisters for filling e-partograph. But their main concern was making another computer available for e-partograph filling.

Some studies showed that training alone cannot support partograph use [25]. However, in contrast to this, our findings show that training along with mentoring and addressing barriers to its use through QI efforts leads to correct and complete use of the partograph.

One of the major limitations of our study was that we did not evaluate whether there was an improvement in maternal and neonatal outcomes with the usage of a partograph. A QI study should be kept simple so as not to overburden the team with the collection of extensive data. Another limitation was that it was not assessed whether the increase in patient load and shortage of trained staff decreased the acceptability of partograph. Larger and adequately powered studies are needed to affirm that the QI methodology can be used to increase the usage of partograph.

## Conclusions

The rate of partograph use increased from 25% to 92% over the study period of six months. QI approach has effectively improved adherence to partograph use, by solving the root cause of the concerns and challenges such as regular training of health care professionals, making department policy, assigning checking authorities, and making triage areas. Simple measures can increase the rate of partograph use. To maintain the results regular audit is necessary.

## References

[1] (2022). World Health Organization. Trends in maternal mortality 2000 to 2017: estimates by WHO, UNICEF, UNFPA, World Bank Group and the United Nations Population Division. https://apps.who.int/iris/handle/10665/327595.

[2] Kieny MP, Evans TG, Scarpetta S (2018). Delivering Quality Health Services: A Global Imperative for Universal Health Coverage. https://reliefweb.int/report/world/delivering-quality-health-services-global-imperative-universal-health-coverage?gclid=CjwKCAjw2OiaBhBSEiwAh2ZSPznodkKd6SBAup7lxFvMh2FhjzAX98m-KVP9qyeaMbik8Z9XZuDYrxoCVJEQAvD_BwE.

[3] Tunçalp Ӧ, Were WM, MacLennan C (2015). Quality of care for pregnant women and newborns-the WHO vision. BJOG.

[4] World Health Organization (2014). WHO Recommendations for Augmentation of Labour. https://www.ncbi.nlm.nih.gov/books/NBK258881/.

[5] Ollerhead E, Osrin D (2014). Barriers to and incentives for achieving partograph use in obstetric practice in low- and middle-income countries: a systematic review. BMC Pregnancy Childbirth.

[6] Fawole AO, Hunyinbo KI, Adekanle DA (2008). Knowledge and utilization of the partograph among obstetric care givers in South West Nigeria. Afr J Reprod Health.

[7] Opiah MM, Ofi AB, Essien EJ, Monjok E (2012). Knowledge and utilization of the partograph among midwives in the Niger Delta Region of Nigeria. Afr J Reprod Health.

[8] Ogwang S, Karyabakabo Z, Rutebemberwa E (2009). Assessment of partogram use during labour in Rujumbura Health sub district, Rukungiri District, Uganda. Afr Health Serv.

[9] Qureshi ZP, Sekadde-Kigondu C, Mutiso SM (2010). Rapid assessment of partograph utilisation in selected maternity units in Kenya. East Afr Med J.

[10] Agan TU, Akpan U, Okokon IB (2014). Assessment of the knowledge and utilization of the partograph among non-physician obstetric care givers in the University of Calabar Teaching Hospital, Calabar, Nigeria. Br J Med Med Res.

[11] Katongole SP, Govule P, Masika MA (2015). Improving partograph documentation and use by health workers of Bwera Hospital: a process improvement research. Int J Nurs Health Sci.

[12] Bajpayee D, Sarin E, Chaudhuri S (2020). Strengthening the use of partograph in high caseload public health facilities in India through an integrated quality improvement approach. Indian J Community Med.

[13] Jain S, Kumar P, Jain M (2021). Increasing adherence to plotting e-partograph: a quality improvement project in a rural maternity hospital in India. BMJ Open Qual.

[14] Puri I, Tadi P (2022). Quality improvement. In: StatPearls [Internet].

[15] Etchells E, Woodcock T (2018). Value of small sample sizes in rapid-cycle quality improvement projects 2: assessing fidelity of implementation for improvement interventions. BMJ Qual Saf.

[16] Paxton A, Maine D, Freedman L, Fry D, Lobis S (2005). The evidence for emergency obstetric care. Int J Gynaecol Obstet.

[17] Kongnyuy EJ, Hofman JJ, van den Broek N (2009). Ensuring effective Essential Obstetric Care in resource poor settings. BJOG.

[18] World Health Organization (2017). Managing Complications in Pregnancy and Childbirth: A Guide for Midwives and Doctors, 2nd Ed.. World Health Organization.

[19] Etchells E, Ho M, Shojania KG (2016). Value of small sample sizes in rapid-cycle quality improvement projects. BMJ Qual Saf.

[20] (2017). LAQSHYA - Labor Room Quality Improvement Initiative. https://nhm.gov.in/New_Updates_2018/NHM_Components/RMNCH_MH_Guidelines/LaQshya-Guidelines.pdf.

[21] Silver SA, Harel Z, McQuillan R (2016). How to begin a quality improvement project. Clin J Am Soc Nephrol.

[22] Brugha R, Varvasovszky Z (2000). Stakeholder analysis: a review. Health Policy Plan.

[23] Rakotonirina J, Randrianantenainjatovo CH, Elyan Edwige Vololonarivelo B, Dorasse R, De Dieu Marie Rakotomanga J, Rakotovao AH (2013). Assessment of the use of partographs in the region of Analamanga. Int J Reprod Contracept Obstet Gynecol.

[24] Yisma E, Dessalegn B, Astatkie A, Fesseha N (2013). Knowledge and utilization of partograph among obstetric care givers in public health institutions of Addis Ababa, Ethiopia. BMC Pregnancy Childbirth.

[25] Bedwell C, Levin K, Pett C, Lavender DT (2017). A realist review of the partograph: when and how does it work for labour monitoring?. BMC Pregnancy Childbirth.

